# Sagittal Band Disruption Following Percutaneous Retrograde Intramedullary Screw Fixation of a Metacarpal Shaft Fracture: A Case Report

**DOI:** 10.7759/cureus.86629

**Published:** 2025-06-23

**Authors:** Stephanie Nulty, James Cardinal, Muaz Mian, Joshua W Hustedt

**Affiliations:** 1 Orthopaedic Surgery, University of Arizona College of Medicine, Phoenix, USA

**Keywords:** hand and wrist surgery, hand trauma surgery, metacarpal fractures, retrograde screw fixation, sagittal band ruptures

## Abstract

Metacarpal fractures may be managed operatively or non-operatively, with malrotation being an indication for operative management. High rates of stiffness have been reported following operative management of metacarpal fractures, and intramedullary screw fixation is a widely accepted treatment that allows early range of motion to help prevent stiffness. While there are some known complications of this treatment modality, there are limited reports of sagittal band disruption. We report the case of a man who presented with sagittal band disruption and ulnar deviation following percutaneous retrograde intramedullary screw fixation of a metacarpal shaft fracture. This report includes details of the patient’s injury, treatment, complication, and follow-up, and urges the awareness of the complication and a reconsideration in the fixation approach and technique.

## Introduction

Metacarpal fractures are a common injury treated by hand surgeons, and account for the second most common hand fracture after phalangeal fractures. These comprise 23% of all forearm and hand fractures treated in the emergency department, with 13.6 metacarpal fractures per 100,000 person-years [1]. These injuries may be managed non-operatively or operatively, with operative indications including bone loss, multiple adjacent fractures, unstable fractures, and rotational malalignment of the digit. Specifically, shortening of more than 5-7 mm in any digit and angulation of more than 20 degrees in the index and middle metacarpals and more than 30 degrees in the ring and small metacarpals warrant surgical consideration [2]. Historically, these fractures were treated with closed reduction and percutaneous pinning, open reduction internal fixation, or external fixation. Plating was associated with stiffness as a primary complication, while pinning carried the risk of infection and loss of alignment of the fracture [3].

Intramedullary screw fixation for metacarpal fractures was described in 1981 by Grundberg et al. [4]. This contemporary approach has been recommended for the treatment of transverse, short oblique, and non-comminuted fractures. This technique offers the benefit of stable fixation without significant dissection [2]. Given the minimally invasive approach, patients can expect earlier return to work and minimal complications [3]. Grundberg et al. described the approach utilizing a long dorsal incision; however, now, a 1.5- to 2-cm incision is recommended, with some surgeons electing to insert this device percutaneously [1,4]. Compared to plate and screw fixation for closed, displaced, extra-articular metacarpal shaft fractures, in one study, intramedullary screws were found to have no difference in total active motion or Disabilities of the Arm, Shoulder, and Hand (DASH) scores [5].

The complication rate of metacarpal intramedullary screw fixation was found to be 2.5%-4.6% [6-9]. The most frequent complication was stiffness, while other noted complications included loss of reduction, screw protrusion, infection, reflex sympathetic dystrophy syndrome, hypertrophic scar, and extensor lag. This procedure is accompanied by a known defect in the metacarpal articular surface; however, the clinical implication of this is not yet known [10]. Urbanschitz et al. proposed a risk of extensor tendon damage; however, sagittal band disruption has not been noted as a complication when this topic was reviewed [8,10]. In this report, we present a case of a male patient who presented with sagittal band disruption and ulnar deviation following percutaneous retrograde intramedullary screw fixation of a metacarpal shaft fracture.

## Case presentation

The patient was a 30-year-old man who visited our clinic for a second opinion in October 2024. His initial injury occurred in December 2021, when he sustained a ground-level fall onto his left closed fist. At that time, he was evaluated at an outside hospital, and a physical examination and radiographic examination were done. He was found to have a fracture of the left third metacarpal. In January of 2022, he underwent operative intervention for this injury at the outside hospital and was treated with percutaneous retrograde metacarpal intramedullary screw fixation.

Upon presentation to our clinic, his first complaint was the ongoing pain. The pain primary affected the left middle finger and occurred when the patient would range the digit, particularly bothersome when making a fist. His second complaint included deformity of the digit. He reported subjective angulation of the left middle finger at resting position. When the patient attempted to make a fist, he reported that the digit hit the other fingers. These symptoms impacted his daily activities that led him to seek help at our clinic. Upon further investigation, the most notable activities that bothered him were typing at work and exercise activities that involved using a bar.

A physical examination yielded no atrophy of the hand. There was visible ulnar deviation of the left long finger, noted in both flexion and extension. On palpation, the patient reported tenderness along the metacarpal that was moderate in intensity. Sensation was intact throughout the entire hand in the ulnar, median, and radial distributions. Range of motion of the hand and wrist were full with the exception of the middle finger. He was able to actively extend the middle finger; however, an extensor lag and ulnar deviation were present with active extension as demonstrated in Video 1.

**Video 1:** Clinical examination

**Figure 1:**
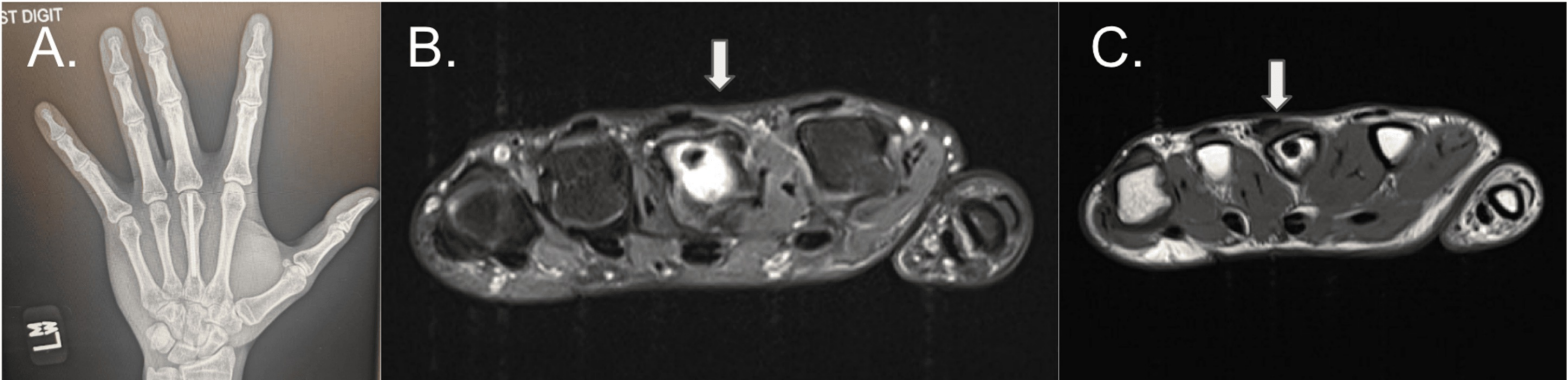
Patient imaging: (A) PA radiograph of the left hand demonstrating percutaneous screw fixation of the third metacarpal. (B) MRI of the left hand demonstrated concern for radial sagittal band disruption and (C) ulnar translation of the extensor tendon of the finger.

Radiographs of the left hand taken in October 2024 demonstrated evidence of percutaneous screw fixation of the third metacarpal with osseous union of the fracture site (Figure 1A). Given the abnormal physical examination findings in the setting of an unremarkable radiograph, the decision was made to pursue advanced imaging. MRI of the left hand was completed in November 2024. A review of these images demonstrated concern for radial sagittal band disruption (Figure 1B). There was visible ulnar translation of the extensor tendon of the finger as well (Figure 1C).

Given the combination of clinical examination and imaging findings, the patient was diagnosed with a sagittal band rupture. Given the history of percutaneous fixation of the third metacarpal fracture, it was hypothesized that the sagittal band was disrupted at the time of percutaneous insertion. The pain and the deformity were both present at the time of presentation to our clinic. There is a possibility that the sagittal band was only partially injured at the index operation, and then later attenuated. The patient reported deformity and pain since the index surgery, making complete injury at the time of the index procedure more likely. The plan for this patient is to undergo sagittal band reconstruction.

## Discussion

Metacarpal fractures are a common injury encountered by hand surgeons, given that they are the third most common upper extremity fractures after distal radius and phalanx fractures [3]. While these injuries were traditionally treated with open reduction internal fixation or closed reduction and percutaneous pinning, intramedullary screw fixation has grown in popularity as a treatment modality.

Given the increased utilization of intramedullary screw fixation for the treatment of metacarpal fractures, it is important for the treating surgeon to be able to recognize and mitigate potential complications associated with this procedure. The complication rate with intramedullary screw fixation of metacarpal fractures has previously been reported to be 2.5%-4.6% [8,9]. This complication rate is relatively favorable in comparison to published complication rates of 10%-32% for plate fixation [11,12] and 16%-41% for pin fixation [13,14]. However, with the comparatively recent introduction of intramedullary screw fixation, it is possible that unique complications such as sagittal band disruption have gone unrecognized.

Sagittal band injuries with extensor tendon instability represent a difficult problem for the patient and the treating surgeon. Though acute sagittal band injuries can be treated non-operatively with splinting, the failure of conservative management is relatively high with late presentation and tendon dislocation predictive of treatment failure [15].

Given the proximity of the extensor mechanism to the insertion site of the intramedullary screw on the metacarpal head, a theoretical risk exists. Urbanschitz et al. conducted a cadaveric study to identify tendon injuries when intramedullary screws were inserted. Screws were inserted into the metacarpal of 32 cadaveric digits in a retrograde fashion, with 16 being inserted percutaneously and 16 being inserted with a mini-open approach [10]. After insertion, dissection was performed to assess injury to the extensor tendon. The group with the mini-open approach had significantly less extensor tendon damage when compared with the group with the percutaneous approach (p < 0.001). Of note, the study additionally investigated the impact of anterograde versus retrograde insertion, finding no difference in tendon damage between the techniques [10]. This highlights the risk to extensor tendons when a percutaneous approach is implemented for metacarpal screw insertion, which was used in our patient's case. Investigation of the sagittal band was not performed in this study; however, it would certainly be of interest for future investigations.

While it is purely speculative to assume that our patient would have benefited from open rather than percutaneous screw fixation, this particular case encourages the discussion regarding the appropriate approach for retrograde screw fixation of metacarpal fractures.

## Conclusions

To our knowledge, this is the first case report to describe an extensor complex disruption following percutaneous intramedullary screw fixation of a metacarpal shaft fracture. Previous reports have provided evidence that percutaneous intramedullary screw fixation can be successfully utilized in metacarpal shaft fractures. However, this case demonstrates that this treatment modality can be problematic as it presents a risk of the complication seen in our patient, which is sagittal band disruption. There is a need for further studies to better understand the incidence of this complication. Nonetheless, our report urges a reconsideration of a percutaneous approach when inserting retrograde intramedullary screws for metacarpal shaft fractures.
